# A microcomputer system for use with an EPR spectrometer

**DOI:** 10.1155/S1463924684000390

**Published:** 1984

**Authors:** M. J. Adams, G. J. Ewen

**Affiliations:** Department of Spectrochemistry The Macaulay Institute for Soil Research Aberdeen AB9 2QJ UK


Journal of Automatic Chemistry, Volume 6, Number 4 (October–December 1984), pages 202–205

### A microcomputer system for use with an EPR spectrometer

**M. J. Adams and G. J. Ewen**

*Department of Spectrochemistry, The Macaulay Institute for Soil Research, Craigiebuckler, Aberdeen AB9 2QJ, UK*

### Introduction

The computing power and data storage capacity of personal microcomputers have increased dramatically during the past few years and this rapid development in semiconductor technology has been reflected in the growing use of microcomputers in analytical laboratories. Important uses for these small computers include the control of instrumentation and the acquisition of data, with subsequent numerical analysis and manipulation of the original data. Although only relatively low-cost devices, these computers, as dedicated systems, can provide a considerable increase in the operating efficiency of complex instrumentation and allow otherwise formidable data-manipulation problems to be undertaken routinely.

At the Macaulay Institute, microcomputer systems are employed with atomic absorption analysis [1] and infra-red spectrometry [2]. The use of a microcomputer interfaced with an EPR spectrometer for spectra acquisition, data manipulation and spectra display on a graphics terminal or, as hard copy, at the spectrometer itself is reported here. The necessary electronic interface circuits between the computer and the spectrometer and graphics unit have been designed and constructed in-house. They comprise 12-bit analogue-to-digital/digital-to-analogue (ADC/DAC) converters and an RS232C asynchronous serial data transfer interface. The computer programs using these interface units have been produced in assembly code as subroutines forming a library for use with PASCAL calling-programs. Typical examples of the operation and performance are discussed and illustrated.

### Instrumentation

The EPR spectrometer is a Model E104-A (Varian Instruments) and, for computer interfacing, is typical of the E-Line EPR Century series of spectrometers. In normal use, a sample spectrum can be recorded directly on the integral flat-bed recorder at a variety of scanning rates and sensitivities. For computer interfacing an external connector (ref. J007) is supplied with the spectrometer. No modification to the spectrometer is necessary to realize the computerized system.

The microcomputer employed is an Apple IIE unit (48 K RAM) with twin $5\frac{1}{4}$ in disk drives and a conventional display monitor. All computer software was written in UCSD PASCAL using the Apple p-code compiler system.

For high-resolution graphic display of spectral data the computer is linked, via an RS232C interface, to a Tektronix 4006 storage graphics monitor.

A schematic diagram of the computerized system is shown in figure 1.

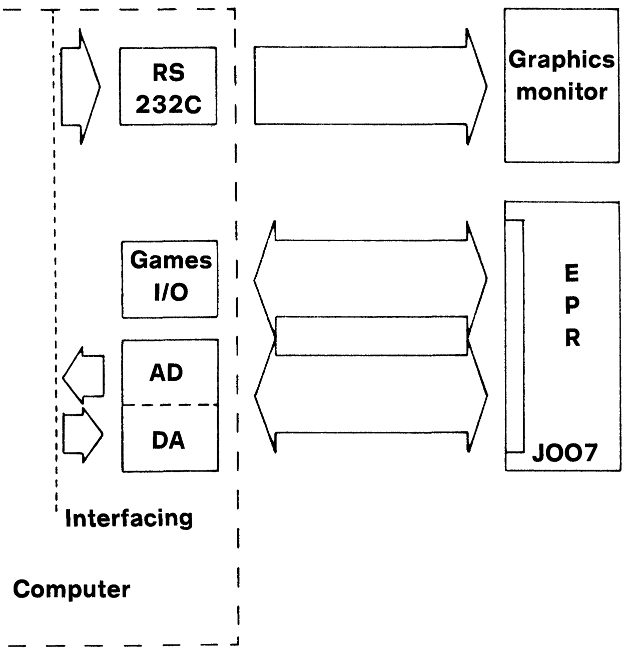

*Figure 1. A schematic diagram of the computerized EPR system illustrating the interface links between the Apple IIE microcomputer and the spectrometer and graphics illustration.*

### *The computer-spectrometer*

The Apple computer to EPR spectrometer connections are illustrated schematically in figure 2. Connector J007, at the rear of the spectrometer, was employed for all the computer to spectrometer links. On this connector, pin A (INT FREQ) provides an output derived from the clock pulse of the stepper-motor drive to the $x$-axis of the spectrometer's recorder at a rate of 10000 pulses per complete scan. This signal is connected to a push-button (TTL) input of the computer game socket, PB-2. A second TTL input port, PB-1, is employed to monitor the status of the left-limit switch of the recorder (pin S). The voltage on this line is low (0 V) before the start of a scan, going high ($\pm 5$ V) during scanning. Whether the spectrometer's recorder plots data from the spectrometer or from the computer is controlled by the status of pin Y on the connector. This was connected to a TTL output, ANO, from the Apple game socket. If held low (0 V) the recorder is isolated from the spectrometer and data from the computer can be plotted directly. The bipolar ($\pm 0{\cdot}5$ V) EPR output data signal is taken from pin X of the connector and connected to the input stage of a $\times 10$ amplifier unit prior to digitizing and computer storage. Digital data from the computer is passed, via a 12-bit DAC, to the spectrometer (pin Z) for plotting. The signal amplifier, ADC and DAC units were designed to provide fast conversion rates and are all contained

© 1984 The Macaulay Institute for Soil Research.

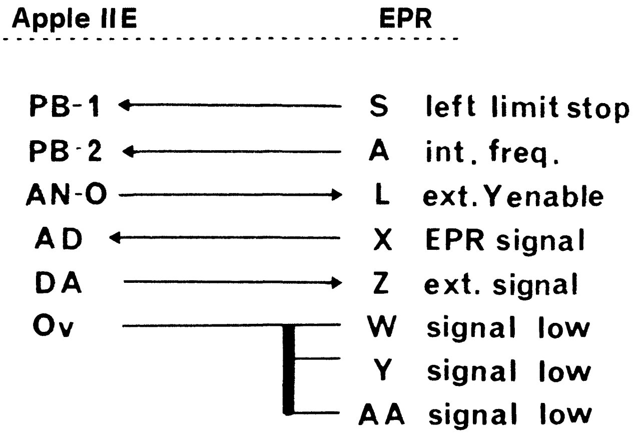

*Figure 2. The microcomputer-EPR spectrometer interface connections employed in the computerized system.*

on a single circuit board which connects directly to one of the input/output edge sockets in the Apple computer. No external power-supply is required. The design and construction details for this interface circuit have been described elsewhere [3]. Briefly, the ADC is based on the CMOS AD574 ic, a precision 12-bit device designed for direct interfacing to microprocessors. Employing the technique of successive approximations, the AD574 can complete a 12-bit data conversion in about 25 $\mu$s.

Digital-to-analogue conversions are achieved with the aid of a 12-bit CMOS AD7542 multiplying DAC. The technical specifications and interfacing notes are available from the manufacturer's literature [4 and 5].

### *The computer-graphics display*

The Apple microcomputer is connected directly with the Tektronix 4006 high-resolution graphics display unit via an RS232C serial interface [6]. The interface is similar to commercially available units and employs a 6850 asynchronous communications interface adapter (ACIA) to perform the necessary parallel-to-serial data conversion for communication between the computer and graphics monitor.

The Tektronix 4006 system provides a high-resolution storage display ($1000 \times 750$ points). A complete spectrum, of 1000 data points, can thus be displayed at the full resolution of the $x$-axis digitization.

### Computer programs

### *Computer-spectrometer*

The transfer of data between the Apple IIE microcomputer and the EPR spectrometer is achieved via the ADC/DAC interface board in an expansion slot (usually No. 4) of the computer, its games socket and the remote connector (J007) on the rear of the spectrometer. For the computer-spectrometer system, three machine-coded subroutines are provided, assembled in a unit library, for use by a main PASCAL program which may call them as external procedures and as an external function. The use and description of these subroutines is briefly described.

*(1) Procedure LINE (STATUS: BOOLEAN)*

The use of this procedure allows the operator to select whether the spectrometer is to operate in its normal, i.e. scanning, mode or to be used simply as an independent flat-bed recorder. To record and acquire spectral data from the spectrometer STATUS should be TRUE.

For plotting computer recorded data it is necessary to disable the plotter from the spectrometer by setting STATUS to FALSE.

Procedure LINE controls the operating mode via a TTL output (ANO) from the Apple game I/O socket.

*(2) Procedure DACONV(DATA: INTEGER)*

This subroutine plots computer stored data on the spectrometer's recorder. To perform the plotting, the routine monitors the pulse of the plotter's $x$-axis stepper-motor and provides the necessary digital-to-analogue conversion. The motor clock pulses are monitored and counted with the aid of switch 2 of the computer's game socket and a new analogue value is output every 10th clock pulse (1000 per spectrum). DATA is an integer value in the range 0-4096 (12-bit resolution) to achieve full-scale ordinate plotting.

*(3) Function ADCONV(DUMMY: INTEGER) INTEGER*

*ADCONV* digitizes an analogue value from the spectrometer with 12-bit resolution, via the ADC interface card, every 10th pulse of the stepper-motor clock. The result is returned as a standard 16-bit integer.

### *Computer-Tektronix display*

Data transfer between the microcomputer and the Tektronix-4006 display unit is achieved via an RS232C asynchronous interface in an expansion slot (usually No. 2) of the Apple. The data-transmission rate is set at 4800 baud, this being the maximum allowed by the graphics unit.

To display EPR data on the Tektronix unit, two machine-coded subroutines are employed.

*(1) Procedure TEKGRAF (DUMMY: INTEGER)*

This subroutine initializes the RS232C interface, clears the graphics screen and sets the display to graphic (plotting) mode. This procedure is called before any graphics data is transmitted and, subsequently, can be used in a program to erase the current display.

*(2) Procedure TEKPLOT (X, Y: INTEGER; MODE: BOOLEAN)*

A 1000-point EPR spectrum can be displayed on the graphics screen. X and Y are the abscissa and ordinate values respectively and should be in the range: $0 < X < 1000$ and $0 < Y < 500$. MODE is a Boolean operator and instructs the display as to whether the X and Y data provided are coordinates for movement only (MODE = FALSE) or movement and plotting (MODE = TRUE).

### Discussion and results

Using the library of simple subroutines, the operator can devise complex data-acquisition and analysis programs in PASCAL or FORTRAN. For example, to achieve a higher signal-to-noise ratio from a sample, multiple scanning with computer signal-averaging can be performed. Figure 3 (*a* and *b*) illustrates this technique [7]. Figure 3 (*a*) is a second-derivative, isotropic fluid solution spectrum of vanadyl histidine at pH 4. This single spectrum was recorded, at room temperature, in 1 min. The spectrum of the same sample recorded 50 times, with automatic

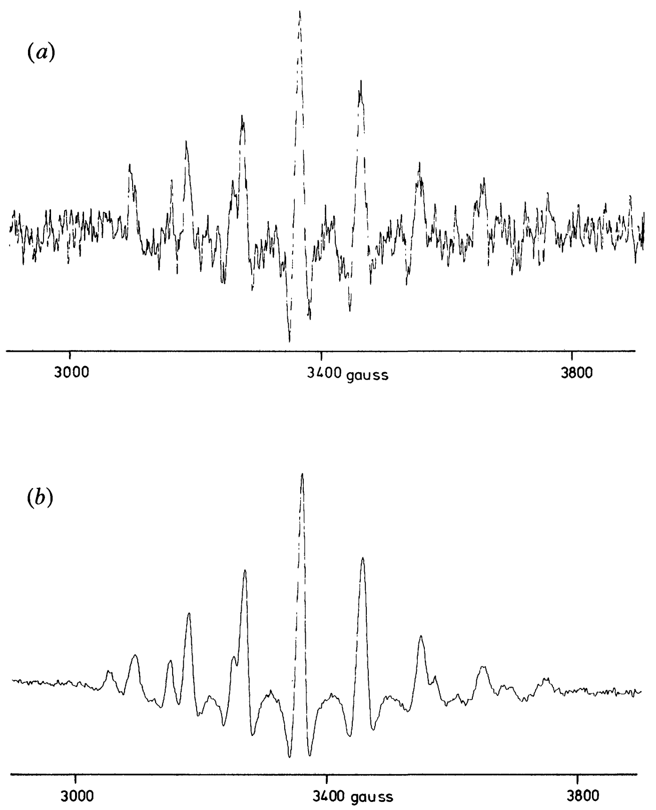

*Figure 3. The signal-to-noise ratio of the spectrum recorded in a single scan (a) is considerably improved if multiple scans are recorded with automatic signal averaging (b).*

signal averaging, is shown in figure 3 (*b*) and demonstrates the presence of two components as a result of the improvement achieved in signal-to-noise ratio. Digital smoothing procedures (moving average, spline fitting etc.) can also be readily employed.

Once the spectral data is digitized and stored in computer-compatible form, many numerical methods are available for manipulating and analysing the data. Figure 4 (*a*) illustrates the first-derivative spectrum obtained from a solution containing a mixture of vanadyl and manganese ions. The pure vanadyl, $VO(H_2O)_5^{2+}$, spectrum is shown in figure 4 (*b*) and, if digitally subtracted from the mixture spectrum, the resultant, figure 4 (*c*), is characteristic of the isolated $Mn(H_2O)_6^{2+}$ spectrum [7].

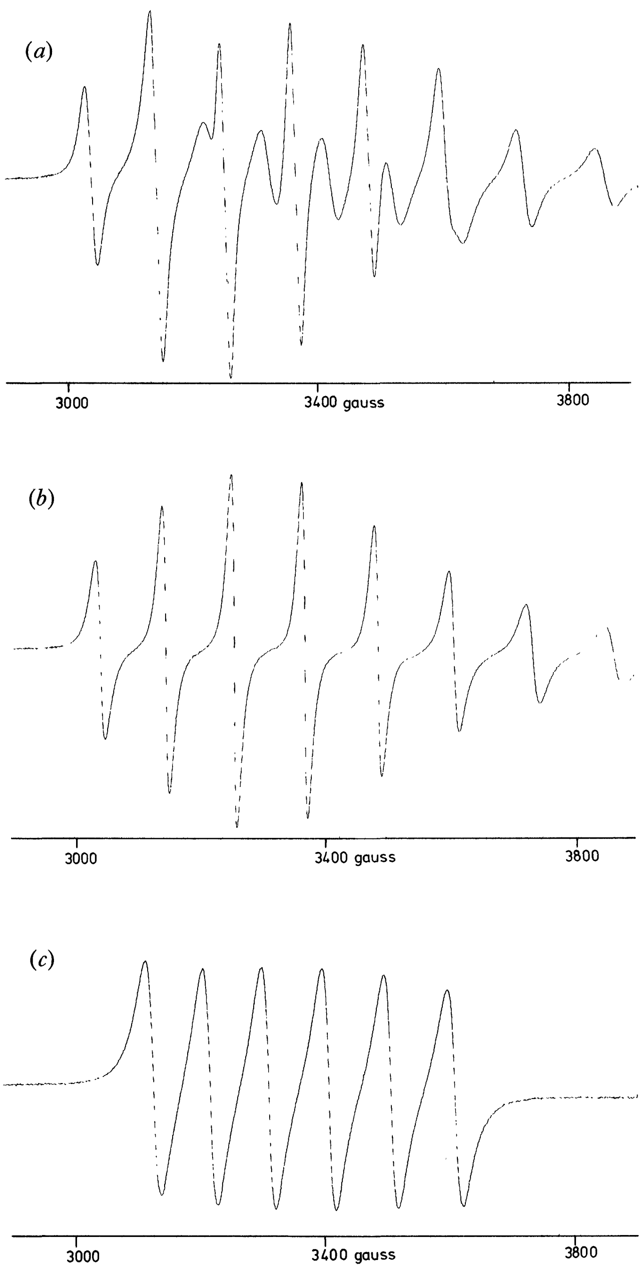

*Figure 4. The difference spectrum (c) can be readily generated from the original mixture (a) by subtraction, with necessary scaling, of one component (b).*

### Conclusions

A microcomputer system has been described which can be readily interfaced to an EPR spectrometer for data acquisition, display and manipulation. The microcomputer employed, an Apple IIE, is a readily available and inexpensive powerful computer with excellent facilities for interfacing to laboratory instrumentation.

The interface circuit boards have been designed and constructed in-house and provide fast and efficient communication between the various instruments in the computerized system.

Although the Apple microcomputer has built-in, medium resolution (280 × 190 points) graphics facilities, the use of an independent graphics monitor, the Tektronix-4006 provides two major advantages. Firstly, the greater resolution of this graphics monitor allows a better and more accurate display of computerized data and, secondly, a great saving in computer memory is achieved. The graphics display of the microcomputer is memory mapped and, hence, memory (approximately 8 Kbytes) must be reserved exclusively for display purposes. This can, in many cases, be considered as a waste of processing memory space and where a graphics terminal is available it can be used to great advantage.

All the control and interface programs for the computerized system have been assembled in PASCAL rather than BASIC, which is a more common microcomputer language. The major advantage of this is that the user is supplied with a library of general-purpose subroutines which can be employed as required, providing for a more flexible operating system. Furthermore, on the Apple microcomputer these PASCAL subroutines can be readily used by programs written in FORTRAN, a computer language more familiar to many scientific workers.

### Acknowledgements

The authors wish to acknowledge the assistance given by D. McPhail in operating the spectrometer and preparing the spectra.

### References

1. Adams, M. J., Mitchell, M. C. and Ewen, G. J., *Analytica Chimica Acta*, **149** (1983), 101.
2. Adams, M. J. and Black, I. J. *Journal of Automatic Chemistry*, **5** (1983), 9.
3. Ewen, G. J. and Adams, M. J. (to be published).
4. Wynne, J. *Analogue Dialogue*, **14** (1980), 9.
5. Timko, M. and Holloway, P., *Analogue Dialogue*, **12** (1978), 3.
6. Ewen, G. J. and Adams, M. J. (to be published).
7. McPail, D. (unpublished work).

### Calendar

### 1985 MEETINGS

**36th Pittsburgh Conference and Exposition on Analytical Chemistry and Applied Spectroscopy**
To be held from 25 February to 1 March 1985 at the Convention Center, New Orleans, Louisiana, USA.
*Further information from David R. Weill III, Shady Side Academy, 423 Fox Chapel Road, Pittsburgh, Pennsylvania 15238, USA.*

**Microprocessor Familiarization**
Course to held from 22 to 23 April 1985 in Bromley, UK.
*Further information from Sira Ltd, South Hill, Chislehurst, Kent BR7 5EH, UK.*

**Health Care—Is it Improved by the Use of Standards in Clinical Laboratory Science? 6th Annual General Meeting and 6th Seminar of the European Committee for Clinical Laboratory Standards**
To be held from 8 to 10 May 1985 at Bornheim-Walberberg, FR Germany.
*Further information from Irene Batty, Executive Director ECCLS, Wellcome Research Laboratories, Langley Court, Beckenham, Kent BR3 3BS, UK.*

**Moisture Measurement in Solids and Gases**
Course to be held from 14 to 15 May 1985 in Chislehurst, UK.
*Further information from Sira (above).*

**Hewlett-Packard 1985 Analytical Symposium**
To be held from 11 to 13 June 1985 at the Wembly Conference Centre, London.
*Further information from Mrs Tina Mears, Hewlett-Packard Ltd, Miller House, The Ring, Bracknell, Berkshire, UK.*

**Product Design Assurance in Engineering**
To be held 11 to 13 June 1985 at the Wembley Conference Centre, London.
*Further information from Mrs H. M. W. Gibbons, Society of Environmental Engineers, Owles Hall, Buntingford, Hertfordshire, UK.*

**Practical UV and Fluorescence Spectrometry**
To be held from 15 to 19 July 1985 in Loughborough, UK.
*Further information from Miss C. D. Newton, Department of Chemistry, Loughborough University of Technology, Loughborough, Leicestershire LE11 3TU, UK.*

**IUPAC 1985**
To be held from 9 to 13 September 1985 in Manchester, UK.
*Further information from the Royal Society of Chemistry, Burlington House, London W1V 0BN.*

**Colloquium Spectroscopium Internationale XXIV**
To be held from 15 to 21 September 1985 in Garmisch-Partenkirchen, FR Germany.
*Further information from CSI XXIV, Organisationsbüro, Institut für Spektrochemie und angewandte Spektroskopie, Postfach 778, D4600 Dortmund 1, FR Germany.*

### 1986 MEETINGS

**4th World Filtration Congress**
To be held in Ostend, Belgium from 22 to 25 April 1986.
*Further information from the Secretariat of World Filtration Congress IV, KVIV-Technologisch Istituut, Jan van Rijswicjcklaan 58, B2018 Antwerpen, Belgium.*

**Analytica 86**
To be held from 3 to 6 June 1986 at München, FR Germany.
*Further information from Münchener Messe- und Ausstellungsgesellschaft mbH, Messegelände, Postfach 12 10 09, D8000 München 12, FR Germany.*

**SAC 86/3rd BNASS: an International Conference on Analytical Chemistry**
To be held from 20–26 July 1986 at the University of Bristol, UK.
*Further information from The Secretary, Analytical Division, Royal Society of Chemistry, Burlington House, London W1V 0BN.*

*Conference organizers whould send full details of their meeting for inclusion in 'Journal of Automatic Chemistry's' calendar at least three months before the event. Information to the Editor, Journal of Automatic Chemistry, Taylor & Francis Ltd, 4 John Street, London WC1N 2ET.*

